# Deep Learning-Enabled Technologies for Bioimage Analysis

**DOI:** 10.3390/mi13020260

**Published:** 2022-02-06

**Authors:** Fazle Rabbi, Sajjad Rahmani Dabbagh, Pelin Angin, Ali Kemal Yetisen, Savas Tasoglu

**Affiliations:** 1 Department of Mechanical Engineering, Koç University, Sariyer, Istanbul 34450, Turkey; fazle.ruet@gmail.com (F.R.); sdabbagh19@ku.edu.tr (S.R.D.); 2 Koç University Arçelik Research Center for Creative Industries (KUAR), Koç University, Sariyer, Istanbul 34450, Turkey; 3 Koc University Is Bank Artificial Intelligence Lab (KUIS AILab), Koç University, Sariyer, Istanbul 34450, Turkey; 4 Department of Computer Engineering, Middle East Technical University, Ankara 06800, Turkey; pangin@ceng.metu.edu.tr; 5 Department of Chemical Engineering, Imperial College London, London SW7 2AZ, UK; a.yetisen@imperial.ac.uk; 6 Institute of Biomedical Engineering, Boğaziçi University, Çengelköy, Istanbul 34684, Turkey; 7 Physical Intelligence Department, Max Planck Institute for Intelligent Systems, 70569 Stuttgart, Germany

**Keywords:** deep learning, machine learning, bioimage quantification, cell morphology classification, cancer diagnosis

## Abstract

Deep learning (DL) is a subfield of machine learning (ML), which has recently demonstrated its potency to significantly improve the quantification and classification workflows in biomedical and clinical applications. Among the end applications profoundly benefitting from DL, cellular morphology quantification is one of the pioneers. Here, we first briefly explain fundamental concepts in DL and then we review some of the emerging DL-enabled applications in cell morphology quantification in the fields of embryology, point-of-care ovulation testing, as a predictive tool for fetal heart pregnancy, cancer diagnostics via classification of cancer histology images, autosomal polycystic kidney disease, and chronic kidney diseases.

## 1. Introduction

Early detection and treatment of illnesses (e.g., cancer) can substantially increase the survival rate, life quality of patients, and, on the other hand, can reduce healthcare-related costs [1,2]. Despite investing a tremendous amount of money in the research and development of diagnostic approaches, the outcome of clinical treatments is not ideal so far [3,4,5]. This problem can stem from the inability of clinicians to acquire enough data, and to analyze healthcare data comprehensively in time [3]. Recent advancements in digital imaging and automated microscopes have led to the creation of copious data at a high pace, addressing the issue of data acquisition for clinicians [1,3,6]. Contemporary automated microscopes, for instance, can produce 10^5^ images per day [7,8]. However, the overwhelming size of the produced data has already outpaced the ability of human experts to efficaciously extract and analyze data in order to make diagnostic decisions accordingly [1,9]. Besides being time-consuming and labor-intensive, human-based analysis can be susceptible to bias [8,10,11]. A combination of modern high throughput clinical methods with the rapidly expanding computational power allows the detection of diseases in a shorter time more accurately, resulting in more robust and accessible health care services for the growing population of the world [9].

Bioimages refer to visual observations of biological processes and structures (stored as digital image data) at various spatiotemporal resolutions. Frequently used techniques in biomedical image analysis are morphology-based cell image analysis, electric signal analysis, and image texture analysis (ranging from single cells to organs and embryos) [12]. For instance, cell morphology, as a decisive aspect of the phenotype of a cell, is critical in the regulation of cell activities [13]. This approach can help clinicians to understand the functionality of various pathogenesis by analyzing the structural behavior of cells [1,12]. Therefore, rapid quantification/analysis of bioimages could pave the path for early detection of disease [14]. However, bioimages exhibit a large variability due to the different possible combinations of imaging modalities and acquisition parameters, sample preparation protocols, and phenotypes of interest, resulting in time-consuming and error-prone analysis by human experts [1,15]. Employing deep learning (DL) techniques can facilitate interpretation of multi-spectral heterogeneous medical data by providing insight for clinicians, contributing to easier identification of high-risk patients with real-time analytics, timely decision making, and optimized care delivery [16,17]. Moreover, DL can support medical decisions made by clinicians, and improve targeted treatment as well as medical treatment surveillance by determination of deviation of the treatment process from the ideal condition [11,18,19,20,21].

DL is significantly contributing to the medical informatics, bioinformatics, and public health sectors. This article provides an overview of DL-enabled technologies in biomedical and clinical applications. We discuss the working principles and outputs of different DL-based applications: architecture models in microfluidics, embryology, point-of-care ovulation testing, as a predictive algorithm for fetal heart pregnancy, cancer diagnostics via classification of cancer histology images, and diagnostic of chronic kidney diseases.

### 1.1. Deep Learning

Machine learning (ML) is a branch of artificial intelligence (AI), which empowers computers to learn using past experiences and example data without being explicitly programmed [22,23]. At a high level, ML algorithms learn to map input feature vectors into an output space, the granularity and data type of which is determined by the particular algorithm used. ML algorithms have been successfully applied in a variety of tasks, including classification, data clustering, time series modeling, and regression. ML methods are broadly categorized as supervised learning algorithms, which utilize labeled data as input to create a model during the training phase, and unsupervised learning algorithms that utilize unlabeled input instances during training. Neural networks are a class of ML algorithms inspired by the human brain, which simulate the encoding, processing, and transmission of information through interconnected neural activities resulting from the excitement or inhibition of neurons in the complex network [23,24].

The foundations of neural networks date back to the 1940s. Hebbian learning rules were introduced in 1949 [25], followed by the first perceptron (1958) [26], the back-propagation algorithm (1974) [27], neocognitron, which was considered as the ancestor of convolutional neural networks (CNNs) (1980) [28,29], Boltzmann machine (1985) [30], recurrent neural network (RNN) (1986) [31], and autoencoders (1987) [32,33]. LeNet, which was the starting point for the era of CNNs, was initially designed for the classification of handwritten digits and reading of zip-code directly from the input without preprocessing (1989) [34]. This was followed by deep belief networks (DBNs) (2006) [31,35], deep Boltzmann machine (2009) [36], and AlexNet, which was the commencement of image classification by CNNs (2012) [31,37,38].

**Figure 1:**
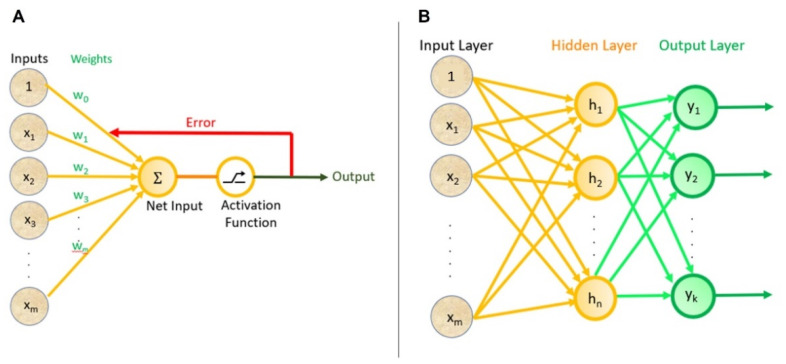
Neural networks. (**A**) The architecture of a perceptron. (**B**) A multi-layer perceptron.

A perceptron, being one of the earliest neural network structures [39], is a linear classifier for binary classifications. A binary classifier is a function that can decide whether an input (i.e., a vector of numbers), fits into a specific class. Perceptron consists of a single input layer directly connected to an output node as shown in Figure 1A, representing the biological process of the human neurons with an activation function and a set of weights [40]. The ML process of a perceptron starts with random weights assigned to each input, which are summed and passed through an activation function that produces an output. The model training process continues with multiple iterations, adjusting the weights, where the ultimate goal is to minimize the total error in the output, i.e., the difference between the output of the model and the actual outputs that should be achieved with the given data instances [41,42].

A multi-layer perceptron (MLP), on the other hand, includes a set of hidden layers between the input and output layers to model more complex networks. While simple perceptron algorithms (i.e., single-layer perceptrons) can learn only linearly separable patterns, MLPs (i.e., feed-forward NNs) possess a greater processing power. A sample MLP containing one hidden layer with *n* nodes and k output nodes is shown in Figure 1B. Here, each input node is connected to each hidden node and each hidden node is connected to each output node, with each edge having weights adjusted during the training process. An MLP can include multiple hidden layers and the hidden layers can consist of varying numbers of nodes. The training process utilizes a back-propagation algorithm [43] that aims to minimize the total error in the outputs of the model by adjusting the weights on the edges in each iteration of the algorithm. The number of input nodes in an MLP is determined by the dimensionality of the input feature vectors, whereas the number of output nodes is decided by the specific ML task. For example, in the case of a regression task, a single output node will be present, whereas, for a classification task, the number of output nodes will be equal to the number of possible classes. In some ML cases, the pattern of data points on the X-Y plane cannot be fully described by a straight line (i.e., a line would not be good enough to predict values) [44,45]. Moreover, when a line is fitted on the data, the output of the function (i.e., predictions) can range from negative infinity to positive infinity (not limited between any ranges). In these cases, non-linear activation functions are a useful tool to remap available data points to a specific range (e.g., between 0 [for highly negative values] to +1 [for highly positive values] for sigmoid function), allowing intentional bending of the regression line (i.e., activation functions are what makes a regression model non-linear to better fit the data) [45,46,47]. Non-linear activation function can result in a more effective and faster algorithm with a lower chance of getting trapped in local minima during training for large/complex datasets with high variety. Typical non-linear activation functions utilized in MLP include sigmoids described by *y*(*v_i_*) = tanh⁡(*v_i_*) and *y*(*v_i_*) = tanh⁡(*v_i_*) + (1 + e^−*v_i_*^)^−1^. The first formula represents a hyperbolic tangent ranging from −1 to +1, while the second equation is the logistic function with a similar shape ranging from 0 to +1. Here, *y*(*v_i_*) is the output of the *i*th node (neuron) and is the weighted sum of the input connections [46].

Early neural networks such as MLP consisted of a limited set of hidden layers (typically 2–3 layers) due to the computational capacities of the machines on which they were trained, confining their modeling ability to simple tasks on well-structured data. With the advances in computer hardware and remote processing capabilities provided by cloud computing, neural networks have evolved into deep neural networks (DNN) containing many more hidden layers allowing for the expression of more complex hypotheses through capturing the non-linear relationships in the network [24]. DL algorithms empower ML to deal with complex multi-dimensional ill-structured data for more real-life applications [23]. DL algorithms utilize multiple layers of artificial neurons to gradually and automatically extract higher-level structures and features from (raw) inputs, including images, videos, and sensor data. Industries, including automotive, aviation, defense, and pharmaceuticals, have recently started to embed DL-enabled technologies into their product development. Training of DL algorithms can be performed with labeled data (supervised learning) for data-driven applications, including face recognition, segmentation, object detection, and image classification [7,48]. On the other hand, unlabeled and unstructured data, which is ubiquitous especially in medical applications, can also be used for the training of DL algorithms (unsupervised learning). Unsupervised DL methods can be used for classification purposes to find structures and similarities among data. DL has revealed superior performance compared to conventional ML methods in many tasks [1,7].

Widely in use DL methods are deep autoencoders, deep Boltzmann machines (DBM), RNNs, DBN, and deep CNN [49]. We describe CNNs in detail below, due to their continued success, especially in automated medical image analysis.

### 1.2. Convolutional Neural Networks (CNN)

DL algorithms including autoencoders, DBN, DBM, and RNN do not scale well in the case of being fed by multi-dimensional input with locally correlated data, as in the case of images [24], which involve huge numbers of nodes and parameters. Convolutional neural networks (CNNs, also known as ConvNet), inspired by the neurobiological model of the visual cortex [50], were proposed to analyze imagery data [51] and became highly successful, forming the basis of many complex automated image analysis tasks today. A CNN is a feed-forward neural network in which signals move in the network without forming loops or cycles [11]. Recently, CNNs have received more attention for medical image analysis and computer vision owing to their ability in extracting task-related features autonomously with no need for human expert intervention, the capability of extracting end-to-end model training parameters by the gradient descent method, and high accuracy [49].

**Figure 2:**
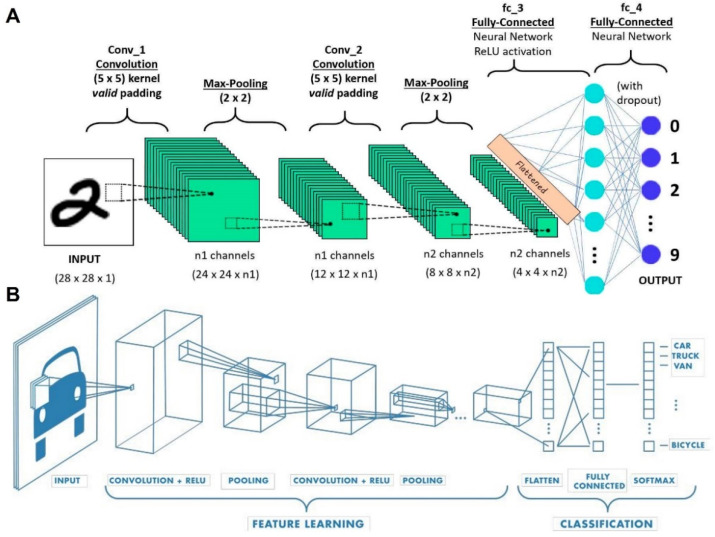
Neural network architectures. (**A**) A convolutional neural network sequence to identify handwritten digits. (**B**) A classic convolutional architecture. Reproduced with permission from [53,54].

CNNs are typically comprised of activation functions, convolutional, pooling, and fully-connected layers [11]. High-level reasonings are done in a fully-connected layer in which neurons are fully connected to all neurons in the previous layer, as seen in Figure 2A,B. The last layer of the fully-connected layer is the loss layer, computing the error as a penalty of the difference between the actual and desired output [38]. Convolution layers perform a linear operation for feature extraction, while a number array (kernel) is applied across the input tensor. To obtain the output value in the output tensor, an element-wise product should be calculated between the input tensor and each element of the kernel [52]. The pooling layer reduces the number of learnable parameters by performing downsampling to decrease the in-plane dimensionality of the feature map [52]. Nonlinearities, which take in a single number and perform mathematical operations, are activation functions. Sigmoid, Tanh, and rectified linear unit (ReLU) are the most commonly used activation functions. The input and output values of Sigmoid are from 0 to 1. Since the outputs of Sigmoid are not zero-centered, gradients oscillate between positive and negative values, which is the main drawback of using Sigmoid with CNNs [38]. Tanh is the scaled-up version of Sigmoid with zero-centered output values ranging from −1 to 1, addressing the abovementioned drawback. However, both Sigmoid and Tanh suffer from the saturation of gradients. ReLU is a linear activation function with a threshold at zero. Applying ReLU can accelerate the convergence of gradient descent in an algorithm [38].

Five popular deep CNNs for feature extraction and classification purposes are AlexNet, visual geometry group network (VGGNet), GoogLeNet, U-Net, and residual network (ResNet) [55]. AlexNet was the first CNN to achieve good performance for object detection and classification purposes [55]. VGGNet and AlexNet are similar networks where VGGNet owns additional convolutional layers. Thirteen convolutional, pooling, rectification, and three fully-connected layers are the constituting layers of VGGNet [56]. However, unlike VGGNet, all convolutional layers are stacked together in AlexNet [38]. GoogLeNet was the first network to implement the Inception module. The Inception module approximates an optimal local sparse structure in a CNN to achieve more efficient computation through dimensionality reduction. The first GoogLeNet was comprised of 22 layers, including rectified linear operation layers, three conventional layers, two fully-connected layers, and pooling layers [38,55]. GoogLeNet possesses fewer parameters compared to AlexNet [38]. U-Net is an architecture with a contracting path and an expansive path, which gives it the U-shaped architecture for semantic segmentation (initially designed for biomedical image segmentation) [57,58,59]. It consists of the repeated application of two 3 × 3 convolutions (unpadded convolutions), each followed by a ReLU and a 2 × 2 max pooling operation with stride 2 for downsampling (i.e., 23 convolutional layers in total) [57]. ResNet displayed acceptable classification performance on the ImageNet dataset. In ResNet, instead of learning on referenced functions, the layers learn residual functions with respect to the received input. Combining multiple-sized convolutional filters, ResNet can reduce required training time with an easier optimization process [38,55,56].

## 2. Deep Learning Applications in Microfluidics

Microfluidics allows for multiplexing biotechnological techniques and enabling applications ranging from single-cell analysis [60,61,62,63,64] to on-chip applications [65,66]. It is commonly used in biomedical and chemical research [67,68,69,70,71,72,73] to transcend traditional techniques with the capability of trapping, aligning, and manipulating single cells for cell combination [74], phenotyping [75,76,77], cell classification [78,79,80,81], and flow-based cytometry [82,83,84], cell capture [85,86], such as circulating tumor cells [87], and cell motility (e.g., sperm movement [88,89], mass [90], and volume sensing [91]). These applications generate high volumes of data of diverse types [92,93]. For instance, a common time-lapse microscopy imaging can create more than 100 GB of data over a day. The advances in DL offer a path to enhance the quality of data analytics when handling large amounts of data such as sequences and images.

**Figure 3:**
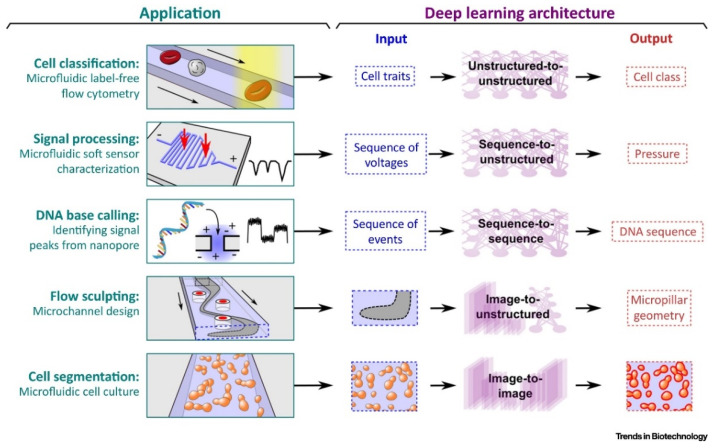
Illustration of applications showing different DL architecture. Reproduced with permission from [101].

Conventional DL algorithms have been paired with microfluidics analysis. This strategy has enabled progress in numerical approaches, including cancer screening [94,95], cell counting [96], and single-cell lipid screening [97]. DNNs have been applied to a wide range of fields, including computational biology [98], biomedicine [23,99], single-molecule science [100]. Architectures used in microfluidic applications can be classified based on the type of input and output data (Figure 3) [101].

Singh et al. [94] presented digital holographic microscopy to identify tumor cells in the blood. The cells were classified according to size, maximum intensity, and mean intensity. The device can detain each cell flowing across a microchannel at 10,000 cells per second. Utilizing ML methods, vigorous gating conditions were established to classify tumor cells in the context of blood cells. As a training set, 100,000 cells were used, and the classifier was made by using the features from those training sets. The resultant area under the curve (AUC) was greater than 0.9. The ML algorithm enabled the examination of approximately 100 cells and 4500 holograms, reaching a yield of 450,000 cells for each sample. Ko et al. [95] applied an ML algorithm to produce an anticipated panel to specify samples extracted from heterogeneous cancer-bearing individuals. A nanofluidic multichannel device was developed to examine raw clinical samples. This device was used to separate exosomes from benign and unhealthy murine and clinical cohorts and contoured the ribonucleic acid (RNA) inside these exosomes. Linear discriminant analysis (LDA) was used to recognize the mRNA profile’s linear relationships that can identify the mice as healthy, tumor, or PanIN. The resulting AUC was 0.5 for healthy vs. PanIN and 0.53 for healthy vs. tumor.

Huang et al. [96] applied DL on a microfluidic device for the blood cell counting process. Two different ML algorithms were compared for computing blood cells, namely Extreme Learning Machine Based Super Resolution (ELMSR) and CNN-Based Super Resolution (CNNSR). The device took a low-resolution image as input and converted it into a high-resolution image as output. The ELM algorithm is a feed-forward neural network with a single input layer, a single-output layer, and a single hidden layer. Alternatively, a CNN was extensively implemented in DL while working with big datasets. Comparing with ELM, CNN can have more than one hidden layer. An advantage of ELM was the creation of weights arbitrarily between the input layer and the hidden layer so that without recursive training, it is tuning-free. When various types of cells need to be trained under distinct qualities, ELMSR is ideal for accelerating the training operation if the number of available images is high. On the other hand, the direct construction of retrieval and integration of patches, as convolutional layers, was the benefit of using CNNSR. For this particular experiment, resolution improving, CNNSR produced 9.5% better results compared to ELMSR.

Guo et al. [97] introduced a high-throughput label-free single-cell screening of lipid-producing microalgal cells using optofluidic time-stretch quantitative phase microscopy. The microscope offers a phase map as well as the opacity of each cell at a high throughput of 10,000 cells/s, allowing precise cell categorization. An ML algorithm was employed to characterize the phase and intensity pictures obtained from the microscopy. After locating the cells, the noise from the background was eliminated. Subsequently, 188 features were chosen from an open-source software named CellProfiler to classify the images. Eventually, binary classification was performed by training a support vector classifier. The accuracy of that classification was 97.85%. The combination of high-throughput quick path interconnected (QPI) and ML was yielded outstanding performance in that the former offers large data for classification while the latter handles large data in an efficient way, improving the precision of cell classification.

Table 1 provides the applications, input and output data type, and examples of widely used architecture models in microfluidic applications. Categorization unstructured data refers to a feature vector, where the order of elements is not critical, whereas structured data refers to a feature vector that needs to preserve the order of elements such as a sequence or image.

## 3. Emerging Deep Learning-Enabled Technologies in Clinical Applications

DL has created highly effective approaches in the biomedical domain, advancing the imaging systems for embryology and point-of-care ovulation testing, predicting fetal heart pregnancy. DL has been used in classifying breast cancer histology, detecting colorectal cancer tissue, and diagnosing different chronic kidney diseases. In this section, a brief description of these emerging DL-enabled technologies in clinical applications is discussed.

### 3.1. Deep Learning-Based Applications in the Field of Embryology and Fertility

#### 3.1.1. Embryology and Ovulation Analysis

**Figure 4:**
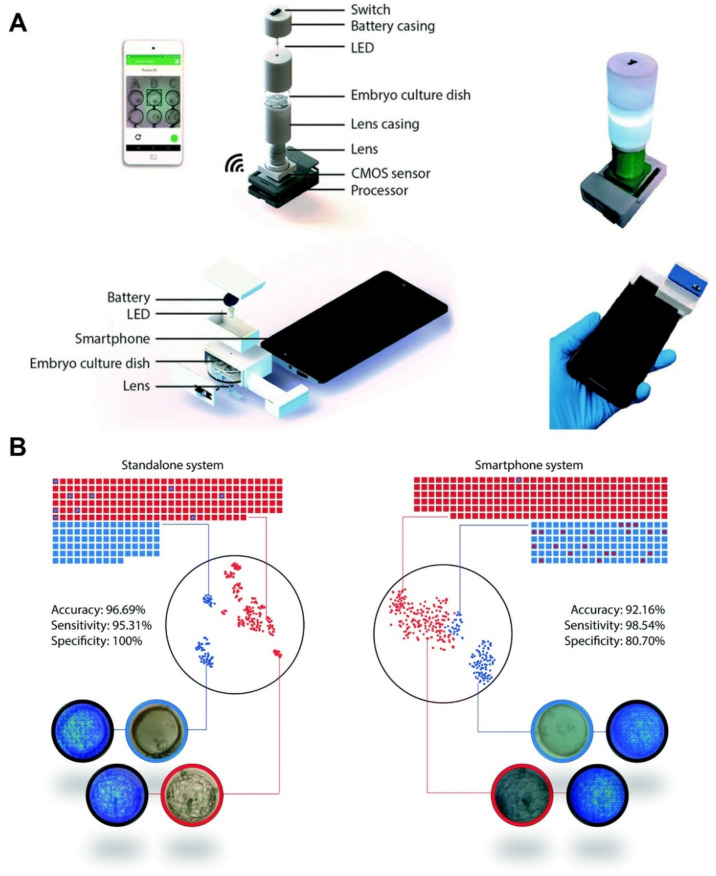
DL-based portable imaging system for embryo assessment and results of the system with pictures taken from stand-alone and cell phone imaging techniques also including a t-SNE and saliency visual analytics. (**A**-top) Autonomous (wireless) imaging device for embryo assessment and the specific parts. (**A**-bottom) Diagram of an embryo imaging device based on a cellphone and its major elements. (**B**-left) The efficiency of the system in assessing imaged embryos using an autonomous device (*n* = 272). (**B**-right) The efficiency of the device when testing cellphone-imaged embryos (*n* = 319). The rectangles depict true marks, and the circles are the classification of the method within them. Blue dots represent non-blastocysts, and red dots show blastocysts. Scatter plots produced by t-SNE are provided to help illustrate the distinction of blastocyst and non-blastocyst embryo pictures taken by (**B**-left) the autonomous module and (**B**-right) the cellphone. Reproduced with permission from [112].

Globally, almost 50 million couples suffer from infertility [108]. In vitro fertilization (IVF) and time-lapse imaging (TPI) are the most widely used methods for embryology; however, they are costly and time-consuming [109,110], even in developed nations [111]. Additional processes of embryo analyses, which entail genotypical and phenotypical assessment, are not cost-effective. A DL method has been developed to resolve these problems by creating two moveable, low-cost (<$100 and <$5) optical methods for human embryo evaluation, utilizing a DNN prepared through a step-by-step transfer learning system (Figure 4A) [112]. First, the algorithm was pretrained with 2450 embryo images with a commercial TPI method. Second, the algorithm was retrained with embryo pictures observed with the moveable optical instruments. The performance evaluation of the device was carried out with 272 test embryo images. The evaluation was achieved using two types of images (blastocytes and non-blastocytes). The precision of the CNN model in categorizing between blastocytes and non-blastocytes pictured with the stand-alone process was 96.69% (Figure 4B) [112].

**Figure 5:**
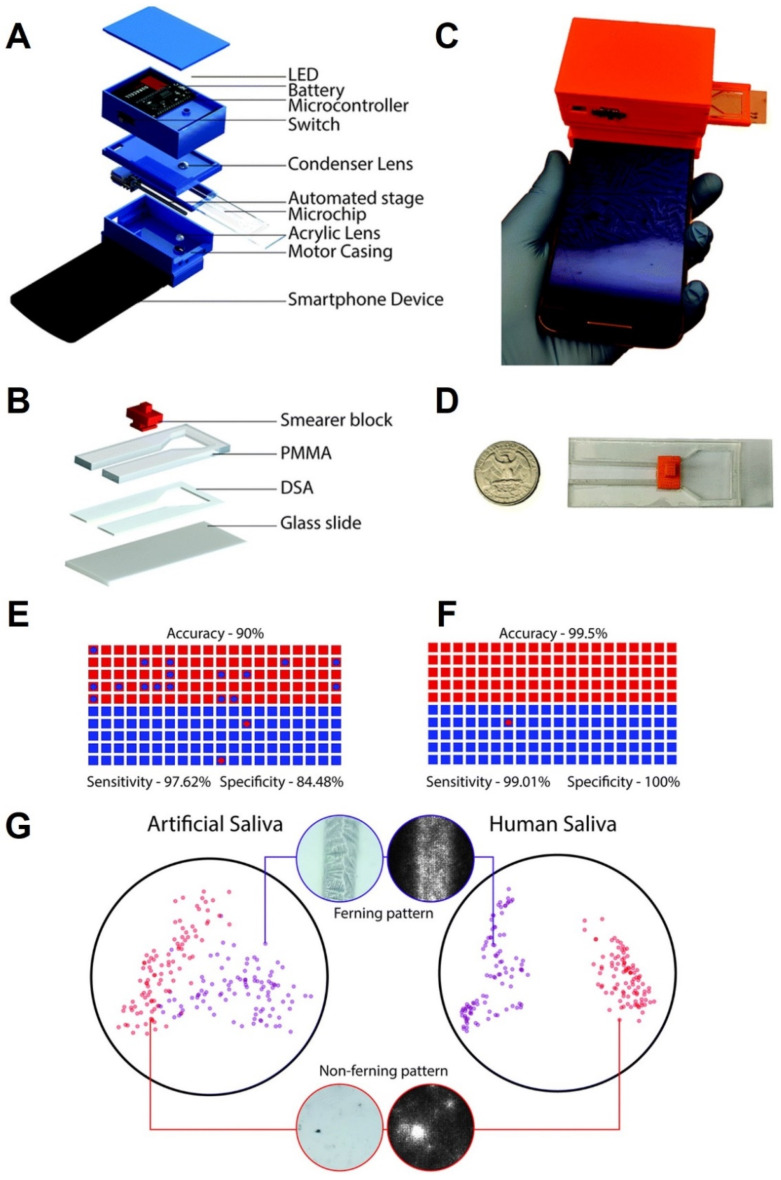
Schematic of the cellphone-based imaging system and reusable microfluidic kit and the system output also with a t-SNE representation with human and artificial saliva samples. (**A**) The view of the autonomous (wireless) optical device and its parts. (**B**) The photograph of the manufactured autonomous imaging method using a smartphone for fern structure imaging and evaluation in naturally dried saliva specimens. (**C**) The schematic of the tool with a holding box. (**D**) A real microfluidic system put near a quarter-coin of US. (**E**) The scatter plot demonstrates the performance of the system in assessing samples of naturally dried unreal saliva (*n* = 200). (**F**) The scatter plot displays the performance of the system when assessing samples of naturally dried human saliva. The rectangles portray true marks, and the circles are the categorization of the scheme. (**G**) The scatter diagram serves to illustrate the distinction of ovulating and non-ovulating types depending on the fern structures shown by the naturally dried human and artificial saliva. Reproduced with permission from [118].

More than 40% of all pregnancies worldwide are unplanned or unintentional [113,114]. Among the different approaches for family planning or pregnancy tests, saliva ferning analysis is relatively simple and low cost [115]. Ferning formations are checked in women ovulating during a 4-day period near the ovulation day [116]. Nevertheless, present ovulation assessments are manual and deeply abstract, resulting in an error when conducted by a lay user [117]. With the help of DL and microfluidic devices, a stand-alone cellphone-based device was developed for point-of-care ovulation assessment (Figure 5) [118]. Nowadays, smartphone-assisted measurements attract more attention due to their low-cost, acceptable detection resolution, and portability [119,120,121,122]. To get rapid and accurate results, a neural network model was run on this device, which completed the process in 31 s. Samples from both artificial saliva and human participants were used to perform the training and testing of the DL algorithm. Thirty-four ovulation specimens ranging from 5.6% to 1.4%, and 30 non-ovulation samples ranging from 0.1% to 1.4% of the synthetic saliva samples were simulated. Lastly, samples of naturally dried saliva were scanned using the optical method based on the cellphone. At total of 1640 pictures of both types of samples were acquired. The pictures were then divided into ovulating pictures (29%), and non-ovulating pictures (71%), depending on the pattern of ferning [118]. A neural network architecture (MobileNet) has been pretrained with 1.4 million pictures from ImageNet to identify the fern structure on a cellphone [123]. ImageNet offers a freely accessible dataset, containing different types of non-saliva pictures. MobileNet’s trained model achieved a top-one precision of 64% and a top-five precision of 85.4% over 1000 ImageNet database classes.

The capability of the MobileNet to anticipate accurate outputs was tested with 100 ferning pattern pictures and 100 without ferning pattern pictures of simulated artificial saliva. The performance of the algorithm in the evaluation of naturally dried saliva specimens was 90% with 95% confidence intervals (84.98–93.78%) (Figure 5E). While analyzing fern patterns of artificial saliva samples, the algorithm acted with a sensitivity of 97.62% (CI: 91.66–99.71%) and a specificity of 84.48% (CI: 76.59–90.54%) (Figure 5E). The positive and negative prognostic values for the test set were 82% and 98%, respectively (Figure 5E). Figure 5G represents a t-SNE diagram for displaying the degree of data divergence in a 2D area, which indicates a strong degree of distinction between the two phenomena. Figure 5F indicates that the precision of the model was 99.5% in anticipating a saliva sample as ovulating or non-ovulating [118].

Bormann et al. [124] designed a DL algorithm for scoring an embryo and compared the output with the results conducted by experienced embryologists. A total of 3469 embryo images were used with two distinct post-insemination (hpi) time periods to train the architecture. Embryo images were divided into five different categories according to their morphology. To examine the embryo scoring, those images were graded by using the model and the embryologists separately. A higher rate of inconsistency was seen among the embryologists while examining the embryos with an average variability rate of more than 82%. However, CNN showed an outstanding result with a 100% recurrence for categorizing the embryo images. Bormann et al. conducted another assessment by selecting the embryo images for biopsy and cryopreservation. For the second task, it was reported that the embryologists picked the embryo images for biopsy with an accuracy of 52%, while the accuracy for the CNN model was 84%. Both results show the supremacy of the DL model for assessing embryology. However, further improvement can be made by enhancing the training facilities of the model.

Chen et al. [125] introduced a DL model for grading embryo images using a “big dataset” of microscopic embryo images. Around 170,000 microscopic images were captured from 16,000 embryos on day 5 or 6 after fertilization. ResNet50 model was used for refining the ImageNet metrics and a CNN was applied to the microscopic embryo images. The labeling of the images was done by using three separate parameters, blastocyte development, inner cell mass (ICM) quality, and trophectoderm (TE) quality. The overall accuracy achieved by the model was 75.3%. Other top-notch research on embryo assessment using a DL network [126] utilized the ANNs model with around 450 images, achieving a precision of 76%. Khosravi et al. [127] designed a DNN using time-lapse photography for continuous automated blastocyte assessment. An accuracy of 98% was achieved in binary classification.

#### 3.1.2. Anticipating the Fetal Heart Pregnancy by Deep Learning

Proper transmission of a single blastocyst will help the mother and child to prevent several adverse medical conditions [128,129]. TPI has a significant impact on valid embryo selection. Since this process requires subjective manual selection, DL provides the possibility for normalization and automation of the embryo selection process. A fully-automated DL model was developed to anticipate the likelihood of fetal heart pregnancy directly from the raw time-lapse videos [130]. This study was conducted in eight different IVF laboratories. Each institute followed its own process of superovulation, egg accumulation, and embryo accumulation. The videos were collected from new embryos, which were fertilized and cultured in a time-lapse incubator for 5 years, and a contemplation analysis was performed. The experiment conducted 1835 different treatments on 1648 patients. The embryos were divided into three categories: multiple transfer cycles (20%), preserved embryos (20%), and fresh embryos (60%).

The performance characteristics of the DL models were evaluated using the receiver operating characteristic (ROC) curve. This curve was produced by plotting the sensitivity against the I-specificity across every possible thresholding value using the anticipated confidence score compared to the actual fetal heart (FH) pregnancy result. Sensitivity and specificity rates could be conducted by selecting a threshold value. A small threshold value will indicate a higher sensitivity with lower specificity and vice versa. The character of this interchange could be evaluated by computing the AUC of the ROC curve. To ensure the robustness of the model, a 5-fold stratified cross-validation was performed [131]. The entire dataset was divided into five equal-sized subsets maintaining the exact ratio of positive embryos. The consequent AUC of the system to anticipate FH pregnancy on the testing dataset was 0.93 with a 95% confidence interval (CI) value, which varied from 0.92 to 0.94. The mean AUC calculated for 5-fold cross-validation was 0.93 [130].

### 3.2. Deep Learning Approaches for Cancer Diagnosis

The treatment of cancers imposes substantial financial burdens on health systems worldwide [132,133]. Breast cancer is the most diagnosed cancer in women worldwide with more than 2 million new cases and an estimated 627,000 deaths in 2018 [132]. In modern cancer treatments, a specific molecular alteration (which can be identified in tumors), is targeted before treatment initiation. The process of visual inspection by a pathologist of biomarker expression on tissue sections from a tumor is a broadly used technique for determining the targeted treatment method. For instance, the semi-quantitative evaluation of the sign of the human epidermal growth factor receptor 2 (HER2), as identified by immunohistochemistry (IHC), indicates the necessity of anti-HER2 therapies for a breast cancer patient. In the case of overexpressed HER2 in the tumor, a treatment against HER2 is more effective compared to chemotherapy alone [134]. Pathologists have reported a considerable variety in diagnostic reports [135,136,137,138,139]; in which 18% of positive cases and 4% of negative cases were misguided [137,140]. The increase in the number of biomarkers will require highly-trained pathologists [141].

To examine the tissues and tumors precisely in a short time, automated diagnosis can be potent for clinical decision-making in personalized oncology. The US food and drug administration (FDA) endorsed the commercial algorithms for computer-aided HER2 scoring [142]. However, despite image analysis-based platforms providing precise IHC biomarker scoring in tumors [138,139], the uses of computerized diagnosis by pathologists have remained restricted. This may be attributed to insufficient proof of clinical significance and the long period needed to specify tumor area in the tissue sample [143]. Recently, DL has been introduced to train computers to identify objects in images [144] of tumors with high accuracy, which will eventually decrease the manual examinations of pathologists. The pathology community is also keen on utilizing DL [145], showing DL-based image analysis can identify cells and categorize cells within distinct cell types [146,147], and find out tumor areas within tissues [148,149]. A further study has been conducted (1) to assess the performance of ConvNets to automatically identify different types of cancer cells and (2) to measure the accuracy of ConvNets to produce precise HER2 condition review in clinical situations.

**Figure 6:**
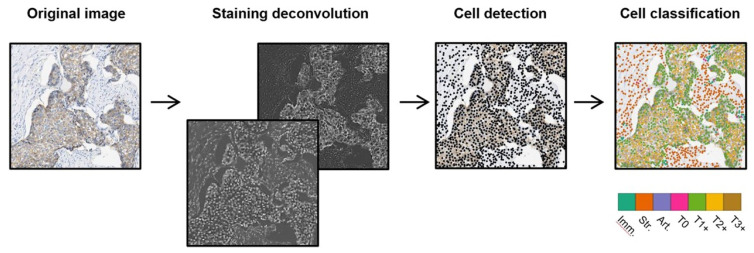
Detection and ranking of tumor cells. Cells are identified using a watershed algorithm and are categorized using DL into seven different types. Reproduced with permission from [142].

Images were analyzed to identify cells, and DL was employed to characterize cells into seven different varieties to score HER2 activity in tumor cells (Figure 6). A total of 74 full-slide photographs of resection samples of breast tumors were obtained from a commercial vendor. After an initial review, 71 carcinoma samples were chosen for further investigation. Then tissues with an automated threshold operation were isolated from the background, and a further phase of color deconvolution was conducted [150] to distinguish these lines for the brown HER2 staining and the blue haematoxyl staining from the actual color picture. HER2 staining and haematoxylin staining networks were uniformly associated with a single photo as a consequence: pixels of a nucleus having a negative value and pixels of positive HER2 membrane staining having positive values. The watershed model [151] was used to divide the tissues into a cell. Conventional ML models were developed to anticipate the type of cell depending on the cell attributes employing architectures in the R programming environment. Based on popularity and high accuracy in several classification tasks [152], linear support vector machine (LSVM) [153], and random forests [154] models were selected. The accuracy achieved for hand-crafted features with LSVM was 68%, for hand-crafted features with random forests was 70%, and for ConvNets was 78%.

**Figure 7:**
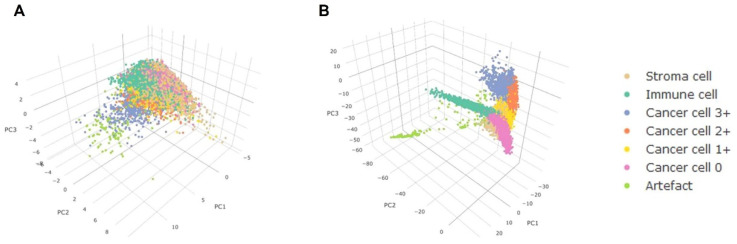
Principal Factor Review of the learned and hand-designed features. The point diagram displays the three primary component values of (**A**) the hand-designed features, and (**B**) the features learned from the convolution neural network. Reproduced with permission from [142].

**Figure 8:**
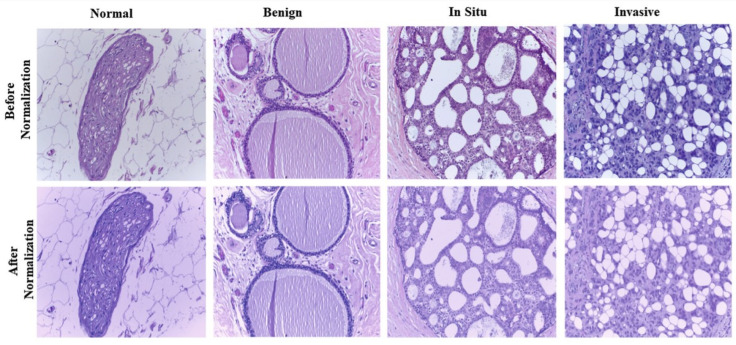
Histology images before and after stain normalization condition. Reproduced with permission from [158].

To comprehend the advantages of ConvNets, principal component analysis was performed to map the hand-crafted high-dimensional aspects, and the ConvNets developed characters through a dynamic 3D environment. Figure 7 shows that the cells in the ConvNets trained feature space are mostly segregated by phenotype while the cells with different phenotypes overlapped in the hand-crafted feature area more. DL has been used in the diagnosis of breast cancer. The diagnosis of tissue growth in breast cancer is made based on primary spotting through palpation and routine check-ups using mammography imaging [155,156]. A pathologist assesses the condition and differentiates the tissues. This diagnosis process requires a manual assessment by a highly-qualified pathologist. A CNN model was designed for the analysis of breast cancer images, which eventually helped pathologists to make decisions more precisely and quickly [155]. To design the algorithm, a dataset of images was composed with high resolution, decompressed, and annotated H&E stain pictures from the Bioimaging 2015 breast histology classification challenge [155]. Four categories of 200× magnified images were classified with the help of a pathologist. A total of 249 images were used to compose the training set, while the test set consisted of 20 images to design the CNN architecture. Preprocessing was performed to normalize the images [157]. Two images are shown in Figure 8 before and after the normalization of the images. CNNs were used to assign the image patches into distinct tissue classes (a) normal tissue, (b) benign tissue, (c) in situ carcinoma, and (d) carcinoma. The accuracy of this method was 66.7% for four classes [155]. The accuracy was 81% for binary carcinoma or non-carcinoma classification [155].

A multi-task DL (MTDL) was used to solve the data insufficiency issue in cancer diagnosis [159]. Although gene expression data are widely in use to develop DL methods for cancer classification, a number of tumors have insufficient gene expression, leading to the loss of the accuracy of the developed DL algorithm. By setting a shared hidden unit, the proposed MTDL was able to share information across different tasks. Moreover, for faster training compared to the Tanh unit, ReLU was chosen as the activation function, along with is Sigmond function in order to get labels in the output layer. Traditional DNN and Sparse autoencoders were used to evaluate the performance of the proposed MTDL. The available data sets were divided into 10 segments, where nine parts were used for training and one part for testing. It was demonstrated that the MTDL achieved a superior classification performance compared to DNN and Sparse autoencoder with smaller standard deviation in results, pointing out a more stable performance [159].

A novel multi-view CNN with multi-task learning (MTL) was utilized to develop a clinical decision support system to specify mammograms that can be correctly classified by the algorithm and those which require radiologist reading for the final decision. Using the proposed method, the number of radiologist readings was reduced by 42.8%, augmenting detection speed, and saving time as well as money [160].

A deep transfer learning computer-aided diagnosis (CADx) method is used for the treatment of breast cancer using multiparametric magnetic resonance imaging (mpMRI) [161]. Features of dynamic contrast-enhanced (DCE)-MRI sequence and T2-weighted (T2W) MRI sequence were extracted using a pre-trained CNN with 3-channel (red, green, and blue [RGB]) input images. The extracted features were used to train a support vector machine (SVM) classifier to distinguish between malignant and benign lesions. The SVM classifier was chosen because SVMs were able to yield acceptable performance on sparse high-dimensional data. Using ROC analysis, the performance of the classifier was evaluated by serving the area under the ROC curve as the figure of merit. The AUCs of 0.85 and 0.78 were reported in a single-sequence classifier for DCE and T2W, respectively, demonstrating the superiority of the purposed system for the classification of breast cancer [161].

In another study, CNNs, including AlexNet, VGG 16, ResNet−50, Inception-BN, and GoogleLeNet, were used for CADx application [55]. Two different methodologies were used for the training of CNNs: (i) fine-tuning, in which weights of the network were previously pre-trained using ImageNet dataset; and (ii) from scratch, in which weights of the network are initialized from a random distribution. While the convergence of all network parameters in (ii) took more time compared to (i), increasing the depth of the network brought about a better ability of discrimination. The fine-tuning method is simpler since most of the corrections of network parameters are applied to the last layers. The maximum performance was reported for ResNet-50 using fine-tuning [55].

In another study, transfer learning was integrated with CNN to classify breast cancer cases [162]. GoogleLeNet, VGGNet, and ResNet, as three different CNN architectures, were used individually to pre-train the proposed framework. Subsequently, using transfer learning, the learning data was transferred into combined feature extraction. The average classification accuracy of GoogleLeNet, VGGNet, and ResNet were 93.5%, 94.15%, and 94.35%, respectively, whereas the proposed framework yielded 97.525% accuracy [162].

A computational method was developed which receives risk patterns from individual medical records to anticipate the outcome of the patient biopsy, for classification of cervical cancer. By formalizing a new loss function to perform dimensionality reduction as well as classification jointly, the AUC of 0.6875 was reported, outperforming the denoising autoencoder method [163].

Colorectal cancer is the third most common cancer in the United States. Reliable metastases detection is needed to diagnose colon cancer. High-resolution images are needed to distinguish between benign colon tissue, cancerous colon tissue, benign peritoneum, and cancerous peritoneum. To produce these images, confocal laser microscopy (CLM) is used to capture sub-micrometer resolution images [164]. These images are then examined by the pathologists to find out the defected region.

**Figure 9:**
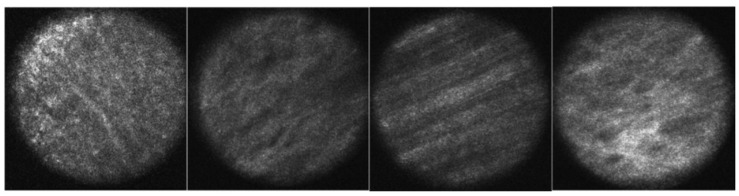
Images of tissue classes in colon cancer. From left to right, benign colon tissue, cancerous colon tissue, benign peritoneum tissue, and cancerous peritoneum tissue. Reproduced with permission from [165].

A method for colon cancer detection was investigated by using DL [165]. Two models, (i) Densenet121 [166] and (ii) SE-Resnext50 [167], were pretrained on the ImageNet dataset. To build the CNN architecture, images of benign colon tissue (*n* = 533), cancerous colon tissue (*n* = 309), benign peritoneum tissue (*n* = 343), and cancerous peritoneum tissue (*n* = 392) (Figure 9) were used. To evaluate the model performance, first, a binary classification was performed to differentiate between the benign colon tissue and benign peritoneum tissue. The highest accuracy for this classification was 90.8% by using Dense TL model. In the next step, to examine the ability to detect the cancerous tissue, the model was tested to classify the benign colon tissue and cancerous colon tissue. For this classification, the model achieved 66.7% accuracy, with a sensitivity of 74.1%. Moreover, the model had an accuracy of 89.1% to classify the benign peritoneum tissue and cancerous peritoneum tissue.

### 3.3. Deep Learning Methodologies in Diagnosing Chronic Kidney Diseases

Chronic kidney disease (CKD), including autosomal polycystic kidney disease (ADPKD) is a public health threat, concerning more than 10 percent of the world’s aged group. It is also regarded among the world’s top 20 causes of death. Recently, DNNs have been used widely to reduce the growth and placate the impact by amplifying the precision of diagnostic methods. For instance, DL is being used in total kidney volume computation on computed tomography (CT) datasets of ADPKD patients. CT and magnetic resonance imaging (MRI) are powerful imaging tools in radiology and biomedical sciences to obtain a snapshot of metabolic changes in the living tissue [168,169]. Additionally, CNN is in use for the semantic segmentation of the MRI for diagnosing ADPKD, as well as detecting CKD from retinal photographs. In this section, applications of DL methods for diagnosing kidney diseases are covered.

Autosomal dominant polycystic kidney disease (ADPKD) is a multisystem genetic condition related to increased kidney volume and expansion of bilateral kidney disease, gradually leading to last-stage kidney disease [170]. In general, Renal Ultrasonography (US) is conducted as a preclinical screening and evaluation of ADPKD for additional initiatives. Different imaging modalities for diagnosis, such as CT and Magnetic Resonance Imaging (MRI), provide higher resolution pictures that assist the detection of subtle cysts [171]. There is a link between total kidney volume (TKV) and kidney function [172], and TKV can be used as an imaging biomarker for predicting malady situations in ADPKD [173,174]. Non-uniform cyst growth increases the variability in kidney morphology, thereby partition of polycystic kidneys for quantifying kidney volume becomes more complicated since the size irregularities are prominent because of the different sizes and shapes of the surface cysts. As a result, an automated segmentation process for accurate TKV measurement remains an ambitious task.

In ADPKD investigation, conventional strategies for total kidney volume calculation dependent on MRI and CT attainments are stereology [175] and manual division. For stereology, each slice is overlaid on a rectangular box with a user indicated cell location and cell separation, and TKV is evaluated by physically listing all boxes surrounding the kidney area. The precision of this approach relies on user-specified variables. The manual partition needs representation of the kidney on each portion using either an accessible hand shaping method or an adaptation to a different technique that manages the user while outlining the subject of concern. CNNs have been suggested for the specificity and differentiation of kidney cells with gentle morphological improvements in medical diagnostics, employing patch-wise strategies on CT [176,177].

Participants were categorized systematically into the testing and training set for the final test, attempting to obtain a comparative allocation in every set based on the usable TKV ranging from 0.321 L to 14.670 L. Two distinct techniques were developed to reduce overfitting and accomplish decent speculation on the training dataset [178]. First, by moving the picture in x–y orientation, and then by distorting the individual slice with non-rigidity and imposing a low-frequency variance in intensity. In the case of the vital analysis, this increases the training data collection almost three times to its previous number. Every one of these data sets is used for the training process to allow it to acquire preferred invariance; for example, shift variance or variable polycystic forms of the kidneys. The slices were mixed before inputting into the CNN. The estimation of output was obtained from the foreground (kidney) and the background (non-kidney) pixels, where pixels with a probability higher than 0.5 were seen as foreground (kidney) pixels.

Baseline and follow-up CT acquisition were 165 training sets and 79 test sets from 125 ADPKD patients, while the TKV ranged from 0.321 L to 1.467 L [178]. Finally, three different types of analysis were performed to summarize the results of this experiment.

**Figure 10:**
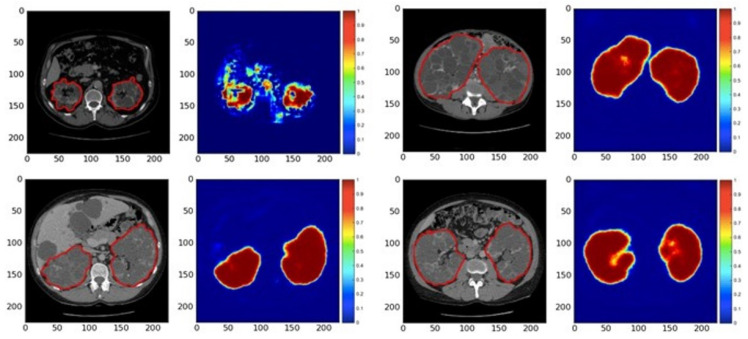
ADPKD Kidney CNN assumptions. Four representations (red contour) of ADPKD kidneys from multiple patient accomplishments. The subsequent CNN-produced graphs are included in pseudo-colors. Reproduced with permission from [178].

**Segmentation Similarity Analysis**: CNN was used for segmentation analysis to produce the output for four patients (Figure 10). This automated segmentation required several seconds [178] for each patient’s CT acquisition, although it took 30 min for each patient to separate manually. The average mean F1 score between automated process classification and ground truth classification from a professional specialist of the kidney was 0.85 ± 0.08 for the entire test set.

**Figure 11:**
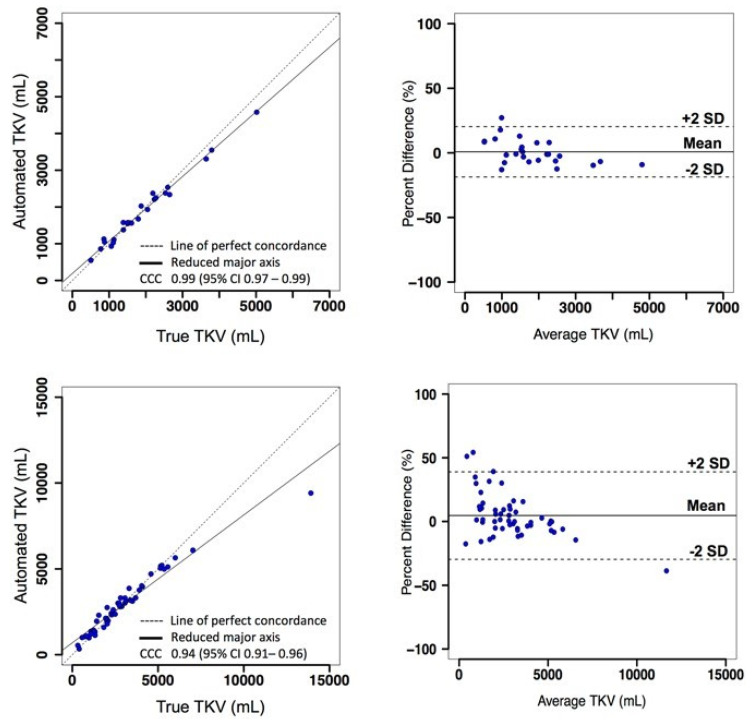
Concordance Correlation Coefficient (CCC) plots demonstrating affiliation intensity (left). Bland–Altman plots display TKV evaluation agreement (right). Reproduced with permission from [178].

**TKV agreement analysis:** A volumetric estimation on kidney differentiation was conducted by using the CNN and contrasted the automated TKV with the actual TKV on the basis of measurement precision [178]. For the first study, there was a generous intensity of the relationship between the automated TKV and real TKV, and the concordance correlation coefficient (CCC) was 0.99, while the confidence interval was 95% (Figure 11 top left). The average TKV deviation between automated and real observations was −32.9± 170.8 mL (*n* = 26 samples), and the average TKV deviation was 1.3 ± 10.3%. Furthermore, Bland Altman plots were used for estimating the collaboration between the two approaches. For the first study, the variances between the minimum and maximum limits of agreement (LOA) were −18.6% and 20.3%, respectively (Figure 11 top right).

For the second and third studies, 53 test cases were performed in combination (Figure 11 bottom left). The real TKV and automated measurements held an average intensity of association of 0.94 CCC. The average TKV deviation between actual and automated measurements was 44.1 ± 694 mL. (Figure 11 bottom right) shows the Bland–Altman plot, where the minimum LOA was −29.6%, and the maximum LOA was 38.9%.

**Cross-Validation Analysis:** To verify the performance from the experimental results, a 3-fold cross-validation was conducted [178]. The Dice Score Coefficients for cross-validation sets were 0.85 ± 0.2, 0.84 ± 0.7, and 0.86 ± 0.5. The mean absolute percentage error varied from 14 to 15%. The Coefficient of variation for all three sets varied from 14 to 15, while the root mean squared percentage error changed from 19 to 21.

Bevilacqua et al. [179] described two different approaches for the semantic segmentation of images that contain polycystic kidneys using CNN algorithms. In the first approach, the whole image was taken as input, without any preprocessing, whereas the second method consisted of two steps. First, a CNN algorithm detected the region of interest automatically, and the semantic segmentation was carried out using the convolutional classifier on the region of interest (ROIs). Multiple topologies were constructed to perform the classification by following the algorithms of SegNet [180] and a fully convolutional network (FCN) [181]. Finally, various matrices, for instance, accuracy and F1 score, were considered to examine the separate classifiers. While the accuracy for the semantic segmentation for the first method was more than 86%, the accuracy for the ROIs classifier was 84%. It is apparent that both methods are equivalent and can be regarded as effective means for the completely automated classification of kidneys impaired by ADPKD when there is a deficit of efficiency in automatic or semi-automatic methodologies, such as function-, atlas-, or model-based strategies.

Subanayagam et al. [182] designed a DL algorithm (DLA) to identify chronic kidney disease using retinal images. Three separate DLAs were developed: (1) using retinal images; (2) considering different risk factors (RF), for instance, age, diabetes, as well as ethnicity; and (3) combining DLA with images and RF. The data for internal validation were taken from the Singapore Epidemiology of eye diseases (SEED) study [183,184,185], and, for the testing of DLAs, two separate datasets were chosen from Singapore prospective study program (SP2) [186], as well as the Beijing eye study (BES) [187]. Approximately 13,000 images were used to train the DLAs, where the DL architecture relied on cCondenseNet [188] with five blocks. Five-fold cross-validation was used to examine the efficiency of the models. The detailed results for the different datasets are shown in Table 2.

To determine the estimated glomerular filtration rate (eGFR) automatically, Kuo et al. [189] proposed a DL algorithm using ultrasound-based kidney images. The neural network was trained by Adam optimizer and optimized by incorporating the robust ResNet model on an ImageNet dataset to predict the function of the kidney. This optimizer is useful to adjust the learning rate automatically for each metric. To anticipate the continuous eGFR, the model gained a correlation of 0.74 with a mean absolute error (MAE) of 17.6 on the testing dataset. In order to classify eGFR with a fixed threshold, the system accomplished an overall precision of 85.6% and area under the ROC of 0.904. The likelihood and efficacy of the model were checked by comparing ResNet-101 model with Inception V4 [190] and VGG-19 [191]. As a result, VGG-19 reduced MAE to 3.1%, while this model demands more sophisticated operations and model sizes compared to ResNet-101.

### 3.4. COVID-19

Coronavirus disease 2019 (COVID-19) rapidly became a global health issue. Radiological imaging of COVID-19 pneumonia revealed the destruction of pulmonary parenchyma, including extensive interstitial and consolidation inflammation which can be used as a means to identify infected people for further treatment. As a result of the COVID-19 outbreak, a large volume of radiological images was obtained daily, outpacing clinicians’ capacity to interpret images. ML has found emerging applications in COVID-19 diagnosis by assisting clinicians to differentiate between COVID-19 and non-COVID19 pneumonia as both COVID-19 and other pneumonia can have similar radiological characteristics [192,193,194,195,196,197]. In this regard, an EfficientNet architecture (consisting of mobile inverted bottleneck MBConv blocks) was developed to classify COVID-19 and non-COVID-19 patients [192]. Classification accuracy of 96% was achieved using a fully connected two-class classification layer (pre-trained on ImageNet). The model was trained using 521 COVID-19 CT images and 665 non–COVID-19 pneumonia images that were split into training, validation, and test sets in a 7:2:1 ratio [192]. In another study, chest X-rays images were classified using a deep two-class classification method, yielding a classification accuracy of 96.34% (between COVID-19 and bacterial pneumonia chest X-rays) and 97.56% (between COVID-19 and non-COVID-19 viral pneumonia chest X-rays) [193]. The training was performed using 130 COVID-19 and 428 non-COVID-19 pneumonia chest X-rays [193]. In order to demonstrate the possibility of implementation of DL-based COVID-19 detection on public datasets, 6868 chest CT images (3102 images labeled as COVID-19-positive and 3766 images labeled as COVID-19-negative) were used to train a ResNet50 CNN algorithm, resulting in a 95.6% accuracy (AUC) on an independent testing dataset [194]. Therefore, ML-assisted COVID-19 diagnosis can facilitate detection of infection in order to take proper action (e.g., isolation and treatment), instead of relying only on human experts to analyze radiological images which is labor-intense, time-consuming, and error-prone.

## 4. Challenges and Concluding Remarks

High-throughput biotechnologies, including microfluidics, could reach a new level of competency by leveraging DL techniques. DL algorithms can find relevant and robust features for the analysis of structured input data (images). This is faster than a human observer capable of extracting a limited set of features or algorithms requiring manual inputs without learning the latent structures in the data. Biotechnology can benefit from DL for analyzing the vast amount of data to predict multifaceted outputs with high accuracy.

The “black-box” issue is one of the main challenges of DL [198]. Although DL (with hidden layers) is a human-designed algorithm, it is not fully understood how these algorithms analyze input data and reach a logical decision inside hidden layers. This issue is not a serious concern in annotating images and voice recognition applications as the user can instantaneously validate the outcome of the DL algorithm to confirm the accuracy as well as the quality of the result. Nonetheless, the black-box issue can cause some concerns in biomedical applications since the employed DL algorithms are inextricably associated with patients’ health (e.g., the DL method can be used to determine the dosage of a drug by receiving the symptoms of the patient as the input data). Lack of transparency on how the DL algorithm determines drug elements can cause a dilemma for both patients and clinicians: whether a patient would be eager to use prescriptions of ML architectures or a clinician should trust the recommended drug as the end product [199]. Moreover, different DL algorithms may suggest different outcomes for the same input data, exacerbating this uncertainty [23,200]. In addition, demanding a large dataset is another challenge of DL considering the fact that in some biomedical fields a limited number of ill people may be willing to participate in clinical research (mainly due to data privacy concerns) [198]. Even with an adequate number of participants and data, disease symptoms and evolution of a known disease can vary from person to person, bringing about uncertainty about the reliability of results of a currently well-performing algorithm for new circumstances.

While existing DL algorithms provide accurate results for classification tasks in the presence of sufficiently labeled data samples for different classes, equally important is the ability to detect occurrences of rare events for which not many data samples exist during training. The ability to accurately detect anomalies in medical data of various types will not only help practitioners identify deviations from the normal state of a patient, but also create opportunities for the diagnosis of rare diseases. As opposed to supervised classification tasks, where the DL models are trained with labeled instances from multiple classes, anomaly detection algorithms are trained with predominantly normal data to detect significant deviations from the normal data they observed during the training process. DL algorithms such as CNN, deep autoencoders [201], long short-term memory (LSTM) networks [202], DBN [203], generative adversarial networks (GAN) [204], and the ensembles of these with classical ML algorithms have been applied for the detection of anomalies in fraud, cyber-intrusion, sensor network anomaly, industrial automation system anomaly, and video surveillance. DL-based anomaly detection also holds significant potential for cell morphology quantification.

Despite their success in classification tasks, classical DL algorithms are usually data-hungry models and do not achieve the same performance when much fewer labeled data samples are used during training. Sufficient training data can be difficult to obtain in some cases due to not only legal restrictions and anonymization requirements, but also the human labor needed to label the data. Recent work in the computer vision community to alleviate this problem has resulted in a class of DL algorithms called one-shot learning models [205], which are capable of learning accurate representations of different classes from even a single training instance. In these cases, where slightly more data instances are available for training, few-shot learning algorithms are utilized. Although popular in the imaging domain so far, these new classes of DL algorithms hold significant potential for application in biomedicine to overcome the difficulties of obtaining a large volume of labeled data. Another method to deal with large unlabeled datasets is the “active learning” method which attempts to maximize the performance of a model while annotating the fewest samples possible [206,207]. In this method, the user initially needs to label a small portion of available data and train the algorithm on that portion (even with low accuracy). Then, the active learning algorithm can prioritize/select a small part of unlabeled data (out of all available data) that needs to be labeled by the user (instead of all available unlabeled data) in order to improve the performance of the training. However, with this method, there is a risk of overwhelming the algorithm with uninformative examples [206,207,208].

With advances in DL, medical diagnostics is expected to experience unprecedented automation in highly accurate detection processes using a variety of data sources. Models that perform a fusion of data from multiple sources will especially provide detailed insights into latent patterns and shape the future of DL-enabled diagnosis.

## Figures and Tables

**Table 1:** Representation of the architecture model in microfluidic applications.

Deep Neural Network Model	Application	Input Parameters	Output Parameters	Example
Unstructured-to-Unstructured	Classify cells using manually processed cell traits	Cell attribute (Perimeter, length of major axis, circularity)	Cell type (colon cancer cell, blood cell)	Cell segmentation and classification with 85% mean accuracy [102]
Sequence-to-Unstructured	Signal processing (evaluate electrical signal to feature the device)	Structured electrical data (sequence of voltage)	Different characterization (pressure at different locations)	Labeling of the soft sensor with 6.2% NRMSE [103]
Sequence-to-Sequence	Monitoring the growth of cell (mass [104] or volume [91]) for a long period of time	A sequence of data (voltage, current)	A classified sequence of data	DNA base calling with 83.2% accuracy [105]
Image-to-Unstructured	Image Processing (detection of lines and edges)	Images	Detection or characterization result of the image	Bacterial growth measuring in a microfluidic system with 0.97 R2 value for deep neural network output [106]
Image-to-Image	Partition of images, anticipating following images in a video	Images	Images with detailed information	Partition of a nerve cell images into different areas with maximum 95% accuracy on mice TEM [107]

**Table 2:** Accuracy and performance of the DLAs for different test sets [182].

	Area Under the Curve (95% CI)	Sensitivity	Specificity	Positive Predictive Value	Negative Predictive Value
**Singapore Epidemiology of Eye Disease**					
Image only	0.91 (0.89–0.93)	0.83	0.83	0.54	0.96
RF only	0.92 (0.89–0.94)	0.82	0.84	0.54	0.95
Hybrid	0.94 (0.92–0.96)	0.84	0.85	0.57	0.96
**Singapore Prospective Study Program**					
Image only	0.73 (0.7–0.77)	0.7	0.7	0.14	0.97
RF only	0.83 (0.8–0.86)	0.73	0.8	0.2	0.98
Hybrid	0.81 (0.78–0.84)	0.74	0.75	0.16	0.98
**Beijing Eye study**					
Image only	0.84 (0.77–0.9)	0.75	0.75	0.09	0.99
RF only	0.89 (0.83–0.95)	0.79	0.82	0.14	0.99
Hybrid	0.86 (0.8–0.9)	0.79	0.79	0.11	0.99
